# *TP53 *mutations, human papilloma virus DNA and inflammation markers in esophageal squamous cell carcinoma from the Rift Valley, a high-incidence area in Kenya

**DOI:** 10.1186/1756-0500-4-469

**Published:** 2011-10-31

**Authors:** Kirtika Patel, Simeon Mining, Johnston Wakhisi, Tarik Gheit, Massimo Tommasino, Ghislaine Martel-Planche, Pierre Hainaut, Behnoush Abedi-Ardekani

**Affiliations:** 1 School of Medicine, Moi University, Eldoret, Kenya; 2 Group of Infections and Cancer Biology, International Agency for Research on Cancer, Lyon, France; 3 Group of Molecular Carcinogenesis, International Agency for Research on Cancer, Lyon, France; 4 Digestive Disease Research Institute, Shariati Hospital, Tehran University of Medical Sciences, Tehran, Iran

## Abstract

**Background:**

Squamous Cell Carcinoma of Esophagus is one of the most common malignancies in both men and women in eastern and south-eastern Africa. In Kenya, clinical observations suggest that this cancer is frequent in the Rift Valley area. However, so far, there has been no report on the molecular characteristics of esophageal squamous cell carcinoma (ESCC) in this area.

**Results:**

We have analyzed *TP53 *mutations, the presence of human papilloma virus (HPV) DNA and expression of inflammation markers Cyclooxygenase 2 (Cox-2) and Nitrotyrosine (NTyR) in 28 cases (13 males and 15 females) of archived ESCC tissues collected at the Moi Teaching and Referral Hospital in Eldoret, Kenya. Eleven mutations were detected in *TP53 *exons 5 to 8 (39%). All ESCC samples were negative for HPV 16, 18, 26, 31, 33, 35, 39, 45, 51, 52, 53, 56, 58, 59, 66, 68, 70, 73 and 82. Immunohistochemical analysis of Cox-2 and NTyR showed a low proportion of positive cases (17.4% and 39.1%, respectively). No association between the above markers and suspected risk factors (alcohol or tobacco use, hot tea drinking, use of charcoal for cooking) was found.

**Conclusion:**

Our findings suggest that mechanisms of esophageal carcinogenesis in eastern Africa might be different from other parts of the world. Low prevalence of *TP53 *mutation compared with other intermediate or high incidence areas of the world highlights this hypothesis. Our data did not support a possible ole of HPV in this series of cases. Further studies are needed to assess and compare the molecular patterns of ESCC from Kenya with those of high-incidence areas such as China or Central Asia.

## Background

Esophageal Squamous Cell Carcinoma (ESCC) is the 6^th ^most common cancer worldwide. About 80% of the cases occur in low and middle income countries, with large geographic variations in incidence [1]. The highest rates (over 50 per 100.000 person-years) are observed in areas of central Asia (in particular Northern Iran and central China). Intermediate incidence rates have been reported for parts of Latin America (Southern Brazil, Uruguay) and several regions in Europe (Western France, Hungary) [2]. The main risk factors in Western Countries include, combined consumption of tobacco and alcohol. In high incidence areas, a number of different factors such as hot tea consumption, low socio-economic status, low fresh fruit and vegetable intake and exposure to dietary carcinogens have been suggested to play a role [3-8]. The involvement of chronic infection by human papilloma viruses (HPV) is a controversial issue. Early results showing a high prevalence of HPV DNA in cases from high incidence areas of China [9,10] have not been confirmed in more stringent, recent studies [11,12].

ESCC has consistently been reported to be a frequent cancer in both males and females in southern and eastern Africa. In South Africa (Transkei region), molecular studies have reported a low prevalence of mutations in exons 5 to 8 of the *TP53 *tumor suppressor gene (17%) [13-16], well below the world average (40%) and the high prevalence observed in high incidence areas of China (70%) [17,18] or Northern Iran (90%) (Abedi-Ardekani *et al*, not published). So far, there is no data on *TP53 *mutation prevalence in ESCC from other parts of Africa.

ESCC is a common cancer in some rural areas of Kenya [19]. In the Rift Valley, ESCC is the leading malignancy in males and the third after cervical and breast cancers in females [20,21]. A recent study in the Bomet District, Western Kenya, showed that ESCC accounted for 914 (34.6%) of the 2643 cancers diagnosed between January 1999 and December 2007, with 6.3% of the patients less than 31 years [22].

We have performed a pilot study on 28 archived, paraffin embedded ESCC tissues from patients referring to the Moi Teaching and Referral Hospital (MTRH, Eldoret, Kenya). We have analysed *TP53 *mutations in exons 5 to 8, presence of high risk types of HPV, and Immunohistochemical (IHC) expression of two markers associated with inflammation damage (NTyR, a direct marker of protein damage by nitric oxide over expressed in ESCC linked with inflammation, [23,24] and inflammatory response (Cox-2, which is often elevated in ESCC from various high-incidence areas, [25]).

## Methods

### Patient selection

Patients who underwent resection for primary ESCC at MTRH, Eldoret, Kenya, between June 2003 and July 2006 were recruited into the study. Diagnosis was performed at the histopathology department, MTRH, and confirmed at the International Agency for Research on Cancer (IARC). A group of 37 properly fixed and processed ESCC samples was assembled for further molecular studies. The patients were part of a case-control study with information on risk factors obtained through questionnaires at the time of diagnosis. All patients involved in that study had signed a written consent. (Patel K. *et al*., unpublished, manuscript in preparation). The study was approved by Ethics Review board of MTRH and of IARC.

### DNA extraction, *TP53 *mutation and HPV analysis

DNA was extracted from areas of tissue sections containing over 50% of cancer cells as identified by a pathologist at IARC (B.A-A) using a column-based purification method. A total of 28 samples provided enough DNA of suitable quality for molecular analyses. *TP53 *mutations in exons 5-8 were analyzed using the IARC reference protocol http://www-p53.iarc.fr/Download/TP53_DirectSequencing_IARC.pdf. Samples were analysed by bidirectional sequencing and the analysis was repeated with two independent PCR products. Sequencing results were compared with the reference sequence X54156 from Genbank http://p53.iarc.fr/TP53sequenceX54156.html. Sequence variations were checked with the mutation validation tool of the IARC *TP53 *mutation database http://www-p53.iarc.fr/MutationValidationCriteria.asp.

HPV typing was performed as previously described [26]. Briefly, detection and typing of 19 mucosal high-risk HPV types (types 16, 18, 26, 31, 33, 35, 39, 45, 51, 52, 53, 56, 58, 59, 66, 68, 70, 73, and 82) was performed by a combination of multiplex PCR and HPV type-specific primers for amplification of viral DNA (E7). PCR products were typed using arrayed primer extension (APEX). Amplification of Beta-globin was used as positive reaction control.

### Immunohistochemistry

Among 28 cases, IHC study was possible on 23. Deparaffinised sections (5 μM) were rehydrated and incubated with primary antibodies for either one hour at room temperature (DO7, mouse-anti p53 monoclonal antibody 1:500, Dako, Glostrup, Denmark), or overnight at 4°C (COX-2, polygoat, 1/2000, Santa Cruz Biotechnology) and NTyR (polyrabbit 1/2000, Upstate Biotechnology). Fixed antibodies were detected with secondary biotinylated anti-rabbit IgG (1/200, Vectasin Elite-ABC kit, Vector Laboratories Inc.) followed by streptavidin-peroxidase and diaminobenzidine-based detection according to standard protocols (Vector Laboratories, Inc.) A score combining intensity of staining (0 to 3) and proportion of stained cancer cells (0-10%: 0; 11-30%: 1; 31-50%: 2; over 50%: 3) was used. The combined scores ranging from 0-9 were scored into 3 final groups: 0-3 (no to weak expression), 4-6 (moderate expression), and 7-9 (strong expression).

## Results

The characteristics of the 28 patients analyzed for both *TP53 *mutations and HPV DNA are summarized in Table 1. This group comprised an approximately equal proportion of males and females, in agreement with the known gender distribution of ESCC in the population of the Rift Valley [21]. Age at diagnosis ranged from 23 to 72 years (average of 56 years) with no difference between genders. All cases were invasive carcinoma.

**Table 1:** Patient's characteristics and suspected risk factors

Patient's characteristics, n = 28		Number
**Gender**	Males	13
	Females	15
**Age**	Average	56.03 ± 12.30
	Range	23-72
**Tobacco use**	Smoking (ever; male/female)	6/0
	Snuff (ever; male/female)	0/7
	Any (ever/never)	13/15
**Alcohol use**	(ever; male/female)	9/1
Hot tea drinking*	(yes; male/female)	6/11
**Consumption of *Mursik***	(yes; male/female)	8/10
**Oral hygiene**^+^	(poor; male/female)	8/8
**Charcoal cooking**	(yes; male/female)	1/12

*: "hot" was considered as immediate drinking after pouring boiling tea

^+^: Estimated on the basis of tooth loss

Among possible risk factors, tobacco was predominantly used as smoked product in males and as snuff in females. Alcohol drinking showed a strong bias towards men (9 males for 1 female). Exposure to charcoal fumes was almost restricted to women (11 of 12 exposed patients were women). Other factors, such as hot tea drinking, consumption of *mursik *(a local fermented milk product laced with charcoal) and poor oral hygiene (as documented by tooth loss) were equally distributed between genders.

*TP53 *mutations (Table 2) were detected in 11 of 28 patients (39.3%). None of these mutations occurred at common *TP53 *mutation hotspots as reported in the IARC *TP53 *database. Of the mutations, 6 were missense, 3 nonsense and 2 were deletions inducing frameshift and supposed to lead to premature termination of translation. Only one mutation was a transversion (A:T to T:A at codon 255, p.I255F). Among transition mutations, 3 were at CpG sites, all leading to nonsense substitutions.

**Table 2:** Characteristics of mutations in Esophageal Squamous Cell Carcinomas from Kenya

Exon	Gene position	cDNA position	Codon and amino-acid change	Mutation	Mutation type
5	g.12524A > G	c.536A > G	p.H179R His-Arg	Missense	A:T > G:C
6	g.12706C > T	c.637C > T	p.R213X Arg-Stop	Nonsense	G:C > A:T at CpG
6	g.12706C > T	c.637C > T	p.R213X Arg-Stop	Nonsense	G:C > A:T at CpG
7	g.13386C > T	c.749C > T	p.P250L Pro-Leu	Missense	G:C > A:T
7	g.13400A > T	c.763A > T	p.I255F Ile-Phe	Missense	A:T > T:A
8	g.13776G > A	c.796G > A	p.G266R Gly-Arg	Missense	G:C > A:T
8	g.13813C > T	c.833C > T	p.P278L Pro-Leu	Missense	G:C > A:T
8	g.13816G > A	c.836G > A	p.G279E Gly-Glu	Missense	G:C > A:T
8	g.13896C > T	c.916C > T	p.R306X Arg-Stop	Nonsense	G:C > A:T at CpG
8	g.13811_13815del5	c.831_835del5	p.?	FS*	-
6	g.12729_12732del4	c.660_663del4	p.?	FS	-

* FS: Frame Shift

Typing of 19 mucosal high-risk HPV types in the same DNA extracts as those used for *TP53 *mutation detection gave negative results (data not shown).

**Figure 1:**
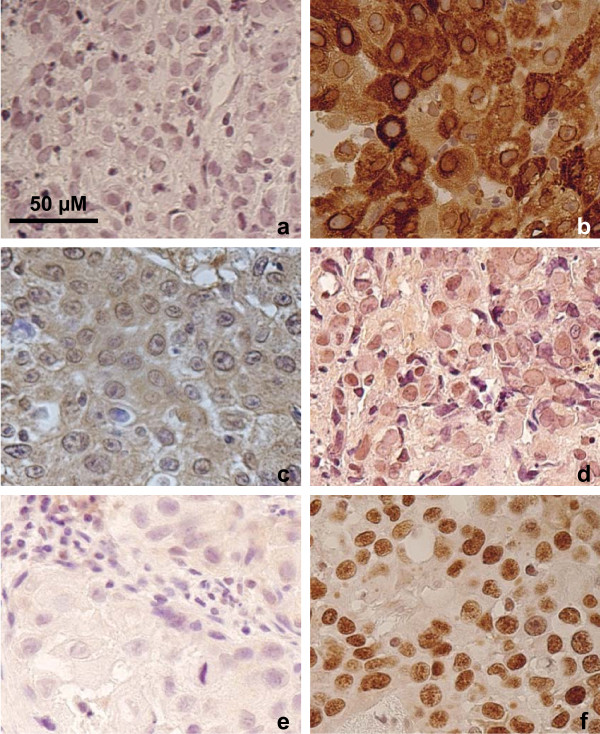
**Detection of Cox-2, N-TyR and p53 proteins by immunohistochemistry in ESCC tissues (×400)**. Typical examples of negative (left panels) or positive (right panels) staining are shown. (a-b): Cox-2; (c-d): N-TyR; (e-f): p53. Bar: scale in μm.

Immunohistochemical analysis on 23 cases (Figure 1 and Table 3) showed p53 protein accumulation (final groups of moderate and strong expression) in 18 (78.2%) of the cases. Among 11 cases with *TP53 *mutations, IHC analysis was possible on 6 and 5 had moderate or strong p53 expression scores. NTyR and Cox-2 were absent or weakly expressed in, respectively, 14 (60.9%) and 19 (82.6%) of the cases, suggesting that expression of markers of inflammation was not a common characteristic of these ESCC cases.

**Table 3:** Expression scores for p53, Cox-2 and NTyR in 23 cases of Esophageal Squamous Cell Carcinomas from Kenya

Expression Score groups	p53 No. (%)	Cox-2 No. (%)	N-Tyrosine No. (%)
Absent or weak (0-3)	5 (21.8)	19 (82.6)	14 (60.9)
Moderate (4-6)	9 (39.1)	3 (13.0)	5 (21.7)
Strong (7-9)	9 (39.1)	1 (4.4)	4 (17.4)

## Discussion

This study is the first report on the molecular characteristics of ESCC from patients of the Rift Valley, a possibly high incidence area in Eastern Africa. Although limited to 28 cases, this series is of interest since there is no report for ESCC in Africa except for a study in Transkei, South Africa [27]. In the present series, men and women were equally represented, a gender distribution similar to most high incidence areas where tobacco and alcohol are not the main risk factors for ESCC, such as in Northern Iran or in central China [2,7,28]. However, from a molecular viewpoint, ESCC from Rift Valley appears different from those high incidence areas in the prevalence of *TP53 *mutations. We report a prevalence of *TP53 *mutation of 39%, far below the high prevalence reported in high incidence areas of China (50-75%) [17,29-32], Northern Iran (90%) (Abedi-Ardekani et al., not published) or Western France (Normandy, 76%) [33]. Remarkably, a low prevalence of *TP53 *mutations has also been reported in Transkei [27]. Furthermore, the mutations identified, both in the present study and in Transkei, differ from the average mutation patterns of most cancers which are dominated by transition mutations at "hotspot" codons within CpG sites [34]. Although the small size of this study precludes any detailed analysis of mutation patterns, these results, together with those previously published for ESCC from Transkei, suggest that ESCC in south-eastern Africa may develop in a different mutagenic context than ESCC in other high-incidence areas. Of note, mutation analysis was limited to exons 5 to 8 since these exons contain about 90% of know mutations in ESCC and were the only exons previously analyzed in ESCC from Africa [27]. This choice was made necessary by the fact that only limited amounts of DNA was available from these formalin-fixed paraffin tissues.

Low prevalence of *TP53 *mutations suggested that other molecular events may take place in ESCC from the Rift Valley. To address this hypothesis, we analyzed the presence of HPV DNA and the expression of markers of inflammation. High-risk HPV are known to contribute to carcinogenesis by inactivating the p53 protein in cervical and in oral cancers, thus bypassing the need for *TP53 *mutation [35,36]. Using a highly sensitive assay for high-risk HPV, we failed to detect any HPV sequences in the 28 cases tested. These results support the conclusions of a previous analysis performed on 29 biopsies of ESCC collected in the Bomet District, Western Kenya [37].

With respect to inflammation markers, only a small proportion of ESCC from the Rift Valley showed positive staining for either NTyR or Cox-2, two markers that we reported as highly expressed in ESCC from Iran (Tehran area) [23]. These results suggest that widespread inflammation is not a characteristic of ESCC in the Rift Valley. However, data on inflammation status of normal mucosa are needed to determine whether inflammatory conditions may play a role in the early steps of esophageal carcinogenesis.

## Conclusions

The results presented here highlight that mechanisms of carcinogenesis in ESCC in eastern Africa may differ from other areas of the world. In particular, the low prevalence of *TP53 *mutation, also observed in ESCC from South Africa, suggests that some other mechanism of p53 inactivation may take place in these cancers. Our data do not support a role for HPV in this process. Furthermore, the risk factors associated with ESCC in Africa is poorly known. In South Africa, fumonisin, a mycotoxin that contaminates several components of the diet, have been proposed as significant risk factors [38,39]. Although fumonisins are present in the diet in east Africa [40], no study so far has addressed their potential role in the etiology of ESCC in the Rift Valley. If further studies with larger sample size and sequencing of all *TP53 *exons confirm our findings, then we may assume that the type of risk factors and/or mechanisms of carcinogenesis in ESCC in Eastern Africa are different from other high-incidence areas of the world.

## Abbreviations

ESCC: esophageal squamous cell carcinoma; HPV: human papilloma virus; Cox-2: Cyclooxygenase 2; NTyR: Nitrotyrosine; MTRH: Moi teaching and referral hospital; IARC: international agency for research on cancer; IHC: immunohistochemistry.

## Competing interests

The authors declare that they have no competing interests.

## Authors' contributions

KP, SM and JW participated in the study concept and provided the archived material. KP participated in the manuscript drafting. GM-P carried out the technical work of molecular analysis and sequencing. TG carried out the HPV DNA analysis. MT supervised the HPV analysis and participated in critical revision of the manuscript. PH was responsible for the study concept, design and supervision, manuscript drafting and critical revision. BA-A was responsible for pathology and immunohistochemistry evaluation, manuscript drafting and critical revision.

All authors read and approved the final manuscript.
